# Non-invasive Biomarkers of Liver Inflammation and Cell Death in Response to Alcohol Detoxification

**DOI:** 10.3389/fphys.2021.678118

**Published:** 2021-07-07

**Authors:** Manuela G. Neuman, Johannes Mueller, Sebastian Mueller

**Affiliations:** ^1^In Vitro Drug Safety and Biotechnology, Department of Pharmacology and Toxicology, Temerity Faculty of Medicine, University of Toronto, Toronto, ON, Canada; ^2^Center for Alcohol Research, University of Heidelberg, Heidelberg, Germany; ^3^Department of Internal Medicine, Salem Medical Center, Heidelberg, Germany

**Keywords:** cytokines, liver stiffness, alcoholic liver disease, apoptosis, inflammation/hepatitis

## Abstract

**Introduction:**

Alcohol-related liver disease (ALD) represents the most common liver disease worldwide, however, the underlying molecular mechanisms are still poorly understood. Namely centrilobular inflammation and programmed cell death are characteristic to ALD and it remains to be elucidated why they persist despite the absence of alcohol.

**Aims:**

To study the effects of alcohol withdrawal in a cohort of heavy drinkers and the role of cirrhosis by using non-invasive biomarkers such as cytokines, apoptotic and angiogenic markers.

**Methods:**

Caspase 3-cleaved M30, M65, cytokines (IL-6, IL-8), tumor necrosis factor alpha (TNF-α), transforming growth factor (TGF-β) and vascular endothelial growth factor (VEGF) were measured in 114 heavy drinkers. The role of alcohol detoxification was investigated in 45 patients. The liver histology was available in 23 patients. Fibrosis stage and steatosis were assessed by measuring liver stiffness (LS) and controlled attenuation parameter (CAP) in all patients using transient elastography (FibroScan, Echosens, Paris). Mean observation interval between the measurements was 5.7 ± 1.4 days (mean + –SD).

**Results:**

Patients consumed a mean of 204 ± 148 g/day alcohol with a heavy drinking duration of 15.3 ± 11.0 years. Mean LS was 20.7 ± 24.4 kPa and mean CAP was 303 ± 51 dB/m. Fibrosis distribution was F0–38.1%, F1-2–31%, F3–7.1 and F4–23.9%. Apoptotic markers M30 and M65 were almost five times above normal. In contrast, TNF- α a, IL-8 and VEGF were only slightly elevated. Patients with manifest liver cirrhosis (F4) had significantly higher levels of M30, M65, IL-6 and IL-8. Histology features such as hepatocyte ballooning, Mallory-Denk bodies, inflammation and fibrosis were all significantly associated with elevated LS, and serum levels of TNF-alpha, M30 and M65 but not with CAP and other cytokines. During alcohol detoxification, LS, transaminases, TGF- β, IL-6, IL-8 and VEGF decreased significantly. In contrast, no significant changes were observed for M30, M65 and TNF- α and M30 even increased during detoxification in non-cirrhotic patients. Profibrogenic cytokine TGF-beta and pro-angiogenic cytokine VEGF showed a delayed decrease in patients with manifest cirrhosis.

**Conclusion:**

Patients with alcohol-related cirrhosis have a pronounced apoptotic activity and a distinct inflammatory response that only partly improves after 1 week of alcohol detoxification. Alcohol withdrawal may represent an important approach to better dissect the underlying mechanisms in the setting of alcohol metabolism.

## Introduction

Alcohol-related liver disease (ALD) is the most common liver disease worldwide ranging from steatosis to liver cirrhosis (Seitz et al., 2018). ALD both depends on various genetic and non-genetic factors including ethnicity (Roerecke et al., 2016) or co-morbidities such as obesity and HCV infection (Mueller et al., 2009; Neuman et al., 2012). In addition, cirrhosis is considered an important pre-cancerogenic lesion ultimately leading to hepatocellular carcinoma (HCC) (Morgan et al., 2004). Most patients with ALD typically present for medical care after developing jaundice or complications of cirrhosis (Zimmerman et al., 1998; Wiesner et al., 2003; Louvet et al., 2017). Given its high prevalence and economic burden, ALD is receiving increasing attention by health authorities and the liver medical and academic communities (Neuman et al., 2001; Yoon and Chen, 2016).

The recent development of non-invasive tools to diagnose various disease stages of ALD by elastography (Mueller et al., 2014), CAP (Thiele et al., 2018), or room temperature susceptometry (Mueller J. et al., 2017) has significantly improved the screening of ALD patients (Moreno et al., 2019). However, optimized non-invasive approaches and refined diagnostic algorithms are still needed to explore promising pharmacological approaches for alcoholic steatohepatitis (ASH), or the rare cases of fatal acute alcoholic hepatitis (AH). Moreover, the therapeutic armamentarium is still limited, and abstinence remains the most effective treatment option in patients with ALD. While studies suggest that early liver transplantation can be successfully performed in highly selected patients with AH its application is limited in most countries due to organ shortage and compliance (Forster et al., 1993; Molina et al., 2002; Neuman et al., 2014a).

The underlying molecular mechanisms of ALD are still incompletely understood. Liver damage due to high alcohol consumption produces a cytokine storm syndrome characterized by the release of pro-inflammatory cytokines. The activation of the immune system is a host defense mechanism (Neuman et al., 2002, 2014b; Neuman, 2007). In response to chronic, heavy alcohol exposure, hepatocytes express and secrete chemokines (Neuman et al., 2017; Le Daré et al., 2019). The role of inflammation in chronic liver disease has also recently lead to a new terminology called acute-on-chronic liver failure (ACLF). Although systemic inflammation is a hallmark of ACLF, its role in the development of this syndrome is poorly understood (Moreau, 2016). Important exogenous inducers further include bacterial products such as pathogen-associated molecular patterns (PAMPs) and virulence factors. Pathogen-associated molecular patterns elicit inflammation *via* innate pattern-recognition receptors (PRRs), whereas virulence factors generally trigger inflammation *via* functional feature identification. Endogenous inducers are called danger-associated molecular patterns (DAMPs) and include molecules released by necrotic cells and products of extracellular matrix breakdown (Neuman et al., 2017; Gao et al., 2019; Gaul et al., 2021).

Increasing evidence suggests an important role for hepatocyte apoptosis in the progression of ALD (Malhi and Gores, 2008; Lavallard et al., 2011), although several other forms of cell death have been described including necrosis, necroptosis, autophagic cell death, and others (Vandenabeele et al., 2010). Both apoptosis and necrosis have also been proposed to be responsible for the development and progression of liver fibrosis (Mehal and Imaeda, 2010). Early during apoptosis, caspases are activated and cleave various substrates including cytokeratin 18 (K18) (Leers et al., 1999; Bantel et al., 2000). K18 is a member of the intermediate filament family of cytoskeletal proteins (Romano et al., 1986). Cytokeratine-generated cleavage fragments of K18 can be detected in serum by the M30 antibody, which specifically labels early apoptotic fragments of cells (Bantel et al., 2000). An increase of apoptotic activity has been demonstrated in heavy drinkers undergoing alcohol detoxification (Mueller S. et al., 2017). This study indicated a link between apoptotic activity and incidence of hepatocellular carcinoma (HCC). The data from detoxification patients suggested that alcohol may inhibit apoptosis and therefore diminishing an important clearance pathway of tumor cells.

Recent studies confirmed the strong association of M30 with steatohepatitis on biopsy in a cohort of patients with severe alcoholic hepatitis (Atkinson et al., 2020). Moreover, in this study, M30 and M65 were associated with 90-day mortality, independent of age and Model for End-stage Liver Disease (MELD). Also, Schlossberger et al. (2019) showed also measured M30 levels in sera of 184 patients with ALD and they did not find significant differences in M30 levels among fibrosis stages and cirrhosis was predicted with a sensitivity of 84.5% and a negative predictive value of 73.5%.

In a cohort of Caucasian heavy drinkers with well characterized disease stages we study the role of alcohol withdrawal and we correlate the clinical information with serum markers of apoptosis and inflammation. In addition, we analyze their association with histological features of ALD in a smaller subcohort. Moreover, we specifically explore the role of cirrhosis in modulating these markers during alcohol withdrawal. Based on proposed role of hepatic arterialization for sustained fibrosis progression (Mueller S., 2016; Piecha et al., 2016), we analyze VEGF to obtain insights on the pro-angiogenic status in the context of inflammation and fibrosis stage.

## Materials and Methods

### Patient Cohorts and Clinical Data

**FIGURE 1:**
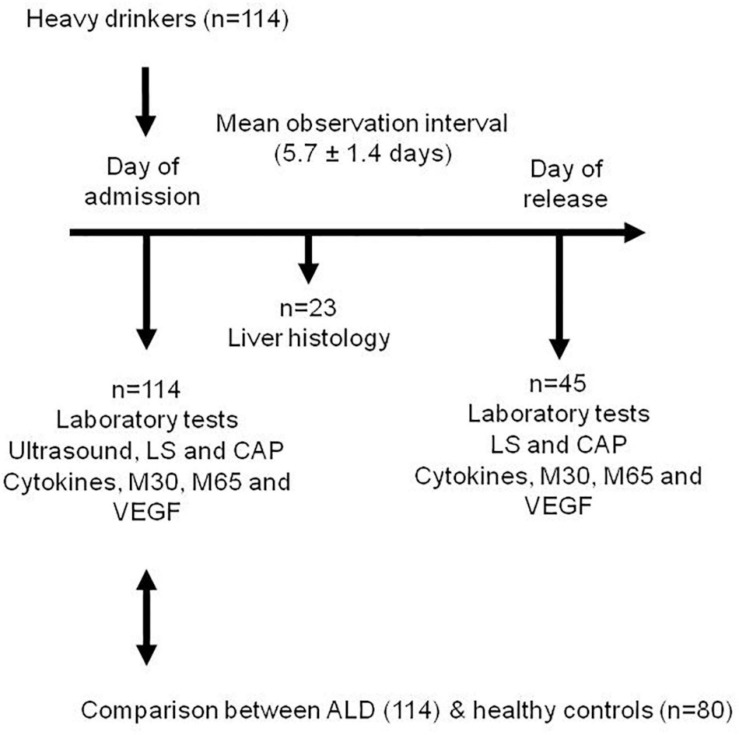
Study design.

The study design is shown in Figure 1.

A total of 114 Caucasian heavy drinkers were prospectively enrolled at Salem Medical Center. All patients with ALD were heavy drinkers (> 80 g per day in males and > 60 g per day in females) with a mean alcohol consumption of 204 ± 148 g/day.

Patient’s characteristics are given in Table 1. All patients were Caucasians with no viral hepatitis B or C (HBV, HCV) infection or human immunodeficiency (HIV) viral infection.

**TABLE 1:** Patient characteristics.

**Parameters**	**ALD**	**Control**	***P*-value**
**General data**			
Number of patients	114	80	
Sex (male)	68%	55%	0.0759
Age (years)	49.9 ± 12.4	34 ± 16	<0.0001
BMI (kg/m^2^)	26.2 ± 5.3	24.0 ± 2.1	0.0005
Alcohol consumption (g/day)	204 ± 148	12 ± 5	<0.0001
Duration of heavy alcohol drinking (years)	15.3 ± 11.0	0	<0.0001
**Ultrasound data**			
Liver size (cm)	16.4 ± 3.7	15.4 ± 2.1	0.0303
Hepatic steatosis (US) (0-3)	1.82 ± 0.86	0.52 ± 0.36	<0.0001
Spleen size (cm)	10.5 ± 2.8	9.9 ± 1.2	0.0730
Presence of Ascites (%)	10%	0%	<0.0001
Signs of cirrhosis (US) (%)	21%	0%	<0.0001
***Elastography data***			
Liver stiffness (kPa)	20.7 ± 24.4	NA	
CAP (dB/m)	303 ± 51	NA	
**Laboratory parameters**			
AST (U/L)	115 ± 101	22 ± 10	<0.0001
ALT (U/L)	74 ± 67	24 ± 15	<0.0001
GGT (U/L)	502 ± 688	33 ± 12	<0.0001
AP(U/L)	115 ± 71	73 ± 21	<0.0001
Bilirubin total (mg/dL)	1.76 ± 3.23	0.3 ± 0.4	<0.0001
INR	1.00 ± 0.27	1.00 ± 0.15	1.0000
Crea (mg/dL)	0.70 ± 0.27	0.72 ± 0.21	0.5795
Hb (g/dL)	13.8 ± 1.9	14.2 ± 1.1	0.0920
Platelets (/nL)	194 ± 81	221 ± 92	0.0320
**Apoptosome and Inflammamosome data**			
M30 (U/L)	566 ± 617	80.0 ± 25.0	<0.0001
M65 (U/L)	1,106 ± 1,040	120.0 ± 60.0	<0.0001
TNF-alpha (pg/mL)	45.6 ± 40.0	90 ± 10.0	<0.0001
TGF-beta (ng/mL)	26.1 ± 23.4	25.0 ± 5.0	0.6796
IL-6 (pg/mL)	47.2 ± 32.9	40.0 ± 15.0	0.0692
IL-8 (pg/mL)	65.9 ± 72.9	40.0 ± 10.0	0.0019
VEGF (pg/mL)	78.0 ± 50.2	60.0 ± 25.0	0.0035

Liver histology data were available in *n* = 23 patients. The study protocol was reviewed and approved by the local Ethics Committee and all patients gave written informed consent prior to inclusion. Other causes of liver diseases were ruled out in all patients serologically by screening for AMA (anti-mitochondrial antibody) and ANA (antinuclear antibody).

Data before and after alcohol detoxification could be obtained in a cohort of 45 individuals. According to the study protocol, blood tests, ultrasound and transient elastography were performed within 24 h after admission and after completion of alcohol detoxification therapy on the day of release from the hospital. Liver biopsies were performed within 48 h after admission.

A cohort of 80 Caucasian healthy individuals served as control group. None of the controls were drinkers or social drinkers. Values for demographic and routine laboratory data are also given in Table 1. All the controls were part of the *In Vitro* Drug Safety and Biotechnology cohort.

### Ultrasound, Transient Elastography (TE) and CAP

Liver size, signs of cirrhosis, spleen size, ascites formation and semi-quantitative liver steatosis (0–3) were assessed by abdominal ultrasound. Liver stiffness was measured (in kPa) using the FibroScan 502 platform (Echosens SA, Paris, France) using both the M and XL probe (Mueller, 2020). Hepatic fat content was also assessed with the Fibroscan device by measuring the controlled attenuation parameter (CAP) (Thiele et al., 2018). CAP values are expressed in dB/m and range from 100 to 400 dB/m. TE was performed by physicians with at least 12 months of experience in abdominal ultrasound and transient elastography on the right lobe of the liver in intercostal position according to established protocols (Mueller, 2020). Fibrosis stages were determined based on the aspartate amino-transferase (AST)-adapted cut-off values as described recently (Mueller et al., 2015).

### Liver Histology

Liver biopsy was available in 23 patients with a mean biopsy lengths of 16.1 ± 12.5 mm). The tissue was fixed in formalin and embedded in paraffin. The histological analysis was performed in 4 μm sections. The tissue was further dewaxed and stained with hematoxylin and eosin (H&E), using standard procedures. Histological scoring was performed as described by Kleiner et al. (2005). The histological diagnosis of steatohepatitis was based on the minimal criteria of steatosis (micro and macro), ballooned hepatocytes, lobular inflammation and Mallory–Denk bodies. In addition, we correlated histological confounders with LS. We also looked at the small nodules (micro-nodular cirrhosis) that surrounded by connective tissue.

### Cytokine Measurements and Apoptosis Markers

Cytokine levels and apoptosis markers were measured at *In Vitro* Drug Safety and Biotechnology, in Toronto, Canada using enzyme-linked immunosorbent-assay (ELISA) with in-house validated controls. Cytokine Kits were as follows: TGF- β (R&D Systems, Inc.; Minneapolis, MN, United States), VEGF, TNF- α levels, IL-6 and IL-8 (pg/mL), (PeproTech Asia, Rehovot, Israel).

The tests were performed according to the manufacturer specifications. For cytokine and apoptosis determination, each specimen was analyzed in duplicate with 95% sensitivity and 92% specificity.

Levels of M30 and M65 were assessed by ELISA using the M30 Apoptosense^®^ ELISA Kit from Bender MedSystems (Vienna, Austria). The procedure is routine in *In Vitro* Drug Safety laboratory. The standards and reference reagents were from the National Institute for Biological Standards and Controls (NIBSC, Herts, United Kingdom).

### Statistical Analysis

For a statistical description of the groups we used mean and standard deviation. Between-group differences were tested for statistical significance using the independent samples *T*-test for continuous variables and the chi-square test for binary data. Change of paired data was tested using the paired samples *T-*test. Correlation analysis was performed using the Spearman’s rank correlation coefficient. *P*-values < 0.05 were considered significant.

## Results

### Patients Characteristics

Patient characteristics are given in Table 1. The heavy drinkers consumed 204 ± 148 g/day alcohol for a duration of 15.3 ± 11.0 years. The liver stiffness (kPa) was 20.7 ± 24.4 (mean ± standard deviation) and CAP was 303 ± 51 dB/m. Mean spleen size was only slightly elevated and ascites was present in 11%.

**FIGURE 2:**
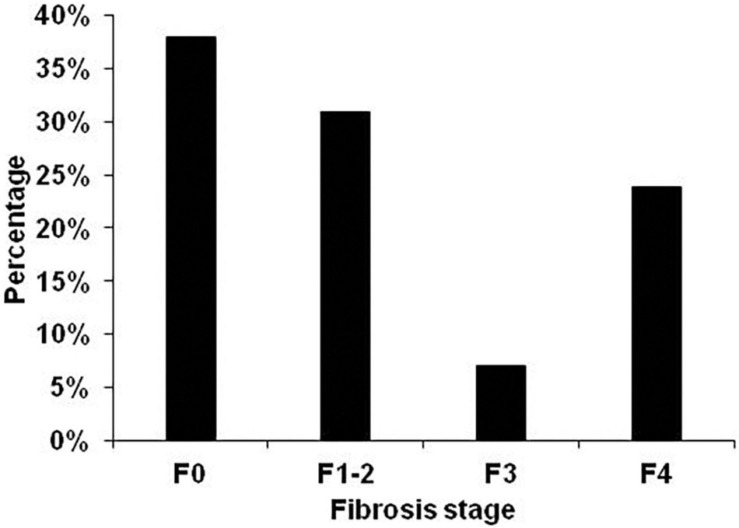
Fibrosis distribution in all 114 patients. Fibrosis stage was non-invasively assessed by transient elastography using AST-adapted cutoff values for liver stiffness (Mueller et al., 2015).

Fibrosis distribution was F0–38.1%, F1–2–31%, F3–7.1% and F4–23.9% (Figure 2).

All laboratory parameters showed a typical profile for a heavy drinker cohort presenting fibrosis and steatosis (Mueller et al., 2014). Consequently, AST, alanine amino-transferase (ALT) and gamma-gluthamyl transpeptidase (GGT) levels were elevated and showed an AST/ALT ratio > 1. Mean total bilirubin was slightly elevated with 1.76 mg/dL. International Normalized Ratio (INR) was normal (1.00). In contrast, apoptotic marker M30 and necrotic marker M65 were five times above normal. Levels of cytokines and chemokines: TNF-α, IL-6, IL-8 and VEGF were only slightly elevated. Correlation analysis is shown in Supplementary Table 1.

LS was significantly correlated with M30 and M65 levels. Serum TNF- α was highly correlated with hemoglobin (*r* = -0.299, *P* < 0.01) and the presence of ascites (*r* = 0.288, *P* < 0.01), TGF- b and IL-8 with bilirubin (*r* = 0.232, *P* < 0.05 and *r* = 0.241, *P* < 0.05), while VEGF correlated highly with hepatic steatosis (*r* = -0.271, *P* < 0.01), platelets (*r* = 0.382, *P* < 0.001) and AST (*r* = -0.307, *P* < 0.001).

In summary, the fibrosis distribution and laboratory parameters of our cohort are representative for a heavy drinker cohort with drastically increased apoptosis markers and slightly elevated cytokines.

### Liver Histology and Inflammation/Apoptosis

The available liver histology from the 23 patients showed the following: Steatosis distribution was: S0 (4.4%), S1 (26%), S2 (17.4%), and S3 (52.2%). Fibrosis distribution was F0 (8.7%) F1 (13.0%), F2 (8.7%), F3 (26.1%), and F4 (43.5%). 82.6% showed ballooning and 87% were classified as steatohepatitis. As shown in Table 2, LS was significantly associated with signs of ballooning, Mallory hyaline bodies, inflammation and fibrosis while no correlation was seen with the degree of steatosis.

**TABLE 2:** Spearman correlations of LS with histological parameters (*n* = 23).

**Histological parameter**	**Correlation with LS**	
	***R***	***p***
Ballooning 0–2	0.741	0.0001
Lobular inflammation 0–3	0.733	0.0001
Classification steatohepatitis 0–2	0.648	0.0011
Fibrosis stage 0–4	0.611	0.0025
Large lipo-granulomas 0–1	0.467	0.0284
Mallory hyaline 0–1	0.451	0.0352
Micro-granulomas 0–1	0.387	0.0756
Microvesicular 0–1	0.362	0.0983
Glycogenated nuclei 0–1	0.346	0.1142
Portal inflammation 0–1	0.303	0.1704
Steatosis grade 0–3	0.298	0.1781
Location 0–3	0.195	0.3845
Pigmented macrophages 0–1	0.131	0.5615
Acidophil bodies 0–1	0.130	0.5653
Megamitochondria 0–1	0.009	0.9698

Correlation analysis of histology with serum markers are shown in Supplementary Table 2.

M30 and M65 correlated with ballooning (*r* = 0.419 and 0.449, *P* < 0.05), lobular inflammation (*r* = 0.506 and 0.489, *P* < 0.05) and Mallory hyaline (*r* = 0.575 and 0.661, *P* < 0.001). TNF-α correlated significantly with hepatic steatosis (*r* = 0.415, *P* < 0.05), lobular inflammation (*r* = 0.441, *P* < 0.05), ballooning (*r* = 0.557, *P* < 0.01), steatohepatitis (*r* = 0.466, *P* < 0.05) and fibrosis septa around the central vein (*r* = 0.487, *P* < 0.05).

IL-6 correlated negatively with steatosis grade (*r* = –0.544, *P* < 0.01), ballooning (*r* = –0.432, *P* < 0.05), acidophil bodies (*r* = –0.440, *P* < 0.05), classification of steatohepatitis (*r* = –0.415, *P* < 0.05) and fibrosis septa around the central vein (*r* = –0.580, *P* < 0.01). No significant correlations were seen for TGF-β, IL-8 and VEGF. Typical ALD-related features of histology correlated highly with LS and apoptosis markers.

### Role of Alcohol Detoxification

In the 45 individuals that underwent detoxification, both LS and CAP decreased significantly over a mean observation interval of 5.7 days in confirmation of earlier reports (Mueller et al., 2010; Thiele et al., 2018). Thus, LS decreased from 32.9 ± 29.7 to 24.8 ± 27.1 (*p* < 0.05) while CAP values declined from 310 ± 50 to 264 ± 77 (*p* < 0.05). In Table 3 we present the changes of laboratory and elastography parameters before and after detoxification.

**TABLE 3:** Laboratory and elastography parameters before and after alcohol detoxification (*n* = 45).

**Parameter**	**Before detox**	**After detox**	***P*-value**
**Laboratory parameter**			
AST (U/L)	134 ± 96	99 ± 107	0.0144
ALT (U/L)	79 ± 74	76 ± 118	0.7748
GGT (U/L)	764 ± 861	453 ± 503	0.0005
AP (U/L)	149 ± 94	117 ± 71	0.0016
Bilirubin total (mg/dL)	2.6 ± 3.89	2.65 ± 4.45	0.8394
INR	1.06 ± 0.27	1.04 ± 0.30	0.8977
Urea (mg/dL)	20.62 ± 12.6	23.1 ± 12.7	0.2230
Creatinine (mg/dL)	0.69 ± 0.29	0.76 ± 0.29	0.0291
Hb (g/dL)	13.1 ± 2.0	13.1 ± 1.9	0.8490
**Serum markers of inflammation and apoptosis**			
M30 (U/L)	811 ± 795	771 ± 626	0.7417
M65 (U/L)	1391 ± 1173	1327 ± 1096	0.5237
TNF-alpha (pg/mL)	56.1 ± 34.9	56.7 ± 95.9	0.9662
TGF-beta (ng/mL)	27.1 ± 31.5	16.7 ± 6.2	0.0280
IL-6 (pg/mL)	43.5 ± 29.6	28.9 ± 13.6	0.0002
IL-8 (pg/mL)	92.6 ± 93.1	60.7 ± 43.3	0.0004
VEGF (pg/mL)	90.8 ± 62.4	61.0 ± 36.0	<0.0001
Transient elastography			
Liver stiffness (kPa)	32.9 ± 29.7	24.8 ± 27.1	0.0421
CAP (dB/m)	310.3 ± 50.03	264 ± 76.7	0.0277

Levels of alanine and aspartate aminotransferases (ALT, AST), alkaline phosphatase (AP) and gamma glutamyl transpeptidase (GGT) also decreased during alcohol withdrawal as described earlier (Mueller et al., 2010). A significant decrease, however, was only observed for AST, GGT and AP but not for ALT and bilirubin. No significant changes were observed for M30, M65 and TNF-α while levels of TGF-beta decreased slightly (*P* < 0.05) or drastically in case of IL-6, IL-8 and VEGF (*P* < 0.001).

We calculated the Spearman correlations of the changes of cytokines during detoxification with other laboratory parameters (Supplementary Tables 3A,B).

The change of M30 was significantly associated with the change in GGT (*r* = 0.367, *P* < 0.05) and absolute hemoglobin levels (*r* = 0.408, *P* < 0.05). The change of M65 was significantly associated with changes in AST (*r* = 0.581, *P* < 0.001), ALT (*r* = 0.529, *P* < 0.01) and GGT (*r* = 0.350, *P* < 0.05) but also absolute GGT levels (*r* = –0.356, *P* < 0.05). The change in IL-8 was significant associated with initial levels of bilirubin (*r* = –0.376, *P* < 0.05) and white blood cells (*r* = –0.333, *P* < 0.05). The change in IL-6 was associated with white blood cells (*r* = 0.329, *P* < 0.05). No relevant associations were seen for VEGF and TGF-β. In conclusion, a significant improvement was observed after 1 week of detoxification for LS, CAP, TGF-β and VEGF but not the other cytokines.

### Role of Cirrhosis Status on Biomarkers During Alcohol Withdrawal

We next studied the role of cirrhosis status on apoptotic markers and cytokines, since cirrhosis itself impairs the immune response, affects programmed cell death and is considered a pre-cancerogenic lesion. Supplementary Table 4 shows comparison between cirrhotic (F4) vs. non-cirrhotic patients (F0-F3) in all 114 patients before detoxification. Cirrhosis was classified according to AST-adapted cutoff-values for LS (Mueller et al., 2015). Cirrhotics were significantly older (54.4 ± 9.1 vs. 48.6 ± 12.9 years, *P* < 0.05), had a significantly longer duration of heavy alcohol drinking (20.1 ± 12.6 vs. 14.3 ± 10.3 years, *P* < 0.05) and had a significantly elevated LS (60.1 ± 14.6 vs. 8.3 ± 8.2 kPa, *P* < 0.001).

Alcohol consumption was slightly decreased in patients with cirrhosis (189 ± 193 vs. 208 134 g/day). They also had significantly higher levels of alcaline phosphatase (AP) (173 ± 94 vs. 95 ± 43, *P* < 0.001), GGT (793 ± 834 vs. 403 ± 613 U/L, *P* < 0.05), bilirubin (3.6 ± 4.3 g/dL, *P* < 0.001) and INR (1.21 ± 0.23 vs. 0.93 ± 0.24, *P* < 0.001) but lowered hemoglobin (12.7 ± 2.2 vs. 14.1 ± 1.7 g/dL, *P* < 0.001) and platelets (161 ± 83 vs. 206 ± 77/nL, *P* < 0.01).

No significant differences were observed with regard to gender distribution, steatosis degree, BMI and levels of ALT and leukocytes. Both M30 and M65 levels were significantly increased in cirrhotics (949 ± 821 vs. 434 ± 473 U/L, *P* < 0.001 for M30 and 1852 ± 1157 vs. 851 ± 864 U/L, *P* < 0.001 for M65). Among the cytokines, only IL-8 was significantly elevated in cirrhotics (97 ± 111 vs. 55 ± 51 pg/mL, *P* < 0.01).

TGF-β and VEGF levels were slightly reduced in patients with established cirrhosis, however, they did not reach levels of significance. The kinetics of cytokine levels, and M30/65 during alcohol withdrawal depends on the cirrhosis status (Table 4).

**TABLE 4:** Biomarkers in cirrhosis (*N* = 18) vs. non-cirrhosis (*N* = 18) before and after alcohol withdrawal.

**Parameter**	**Before**	**After**	**P**
**Non-cirrhotics F0-3 *N* = 26**			
M30 (U/L)	622 ± 654	658 ± 649	0.6581
M65 (U/L)	979 ± 1,019	953 ± 1,101	0.5580
TNF-alpha (pg/mL)	50.4 ± 33.3	40.5 ± 21.4	0.0052
TGF-beta (ng/mL)	30.3 ± 40.4	16.8 ± 6.7	0.0977
IL-6 (pg/mL)	40.5 ± 29.2	25.2 ± 13.6	0.0024
IL-8 (pg/mL)	72.4 ± 65.9	52.1 ± 31.1	0.0197
VEGF (pg/mL)	96.8 ± 69.7	59.4 ± 36.6	0.0002
**Cirrhotics F4 *N* = 18**			
M30 (U/L)	1,046 ± 931	873 ± 583	0.3846
M65 (U/L)	19,02 ± 1,168	1721 ± 898	0.7379
TNF-alpha (pg/mL)	60.4 ± 33.9	46.0 ± 24.6	0.0040
TGF-beta (ng/mL)	23.2 ± 11.2	16.6 ± 5.8	0.0037
IL-6 (pg/mL)	49.7 ± 30.0	34.8 ± 11.9	0.0327
IL-8 (pg/mL)	114.9 ± 118.4	69.5 ± 54.6	0.0124
VEGF (pg/mL)	86.2 ± 50.6	65.3 ± 35.5	0.0023

Cirrhosis was classified using AST-adapted cutoff-values for LS (Mueller et al., 2015).

Pro-fibrogenic cytokine TGF-β decreased significantly in patients with cirrhosis during alcohol detoxification. The levels of TGF-β were identical in cirrhotic and non-cirrhotic individuals after the detoxification period. No significant changes of M30/65 were observed (Table 4).

M30 tended to increase during detox in non-cirrhotics while a decrease was seen in cirrhotic individuals. Despite a smaller cohort size in the cirrhosis subgroup, all changes were slightly significant in response to alcohol withdrawal except VEGF. VEGF remained higher in the cirrhosis group despite the absence of alcohol. Among the biomarkers, only M65 was significantly higher in the cirrhosis group after detoxification.

In conclusion, despite impaired synthesis capacity in patients with cirrhosis, both markers of inflammation and apoptosis were significantly elevated in these patients. Significant reduction of cytokine levels were seen in the cirrhosis group after detoxification.

## Discussion

In a cohort of well characterized heavy drinkers apoptosis. inflammation and vascular activities are mechanistic factors. Apoptosis, as measured by levels of M30, is drastically increased in cirrhotics. Alcohol detoxification for less than 1 week rapidly improved various parameters such as liver stiffness and transaminase levels but not the apoptosis rate. Both markers of inflammation and apoptosis were significantly elevated in these patients.

LS declines significantly in heavy drinkers after only 1 week of alcohol abstinence. This confirms earlier reports (Mueller et al., 2010). This decrease was statistically significant in 45.3% of the patients and resulted in a decrease of the estimated stage of fibrosis in 23.3% of them. LS was also significantly associated with histological features of fibrosis, ballooning and inflammation but not steatosis. The findings that LS was even higher correlated with inflammation than fibrosis is due to smaller sample size in comparison to an earlier report (Mueller and Lackner, 2020). Apoptosis rate was highly associated with histological features of ballooning, lobular inflammation and fibrosis (Stärkel et al., 2019). These data suggested that alcohol has a strong inhibiting effect on cell regeneration and apoptosis while withdrawal will enhance cell regeneration.

In the present study we provide further evidence that M30 levels are generally higher in cirrhotic patients. The increase of M30 in response to withdrawal is only seen in non-cirrhotic patients. These novel findings suggest that patients with cirrhosis have a liver micro- environment that, independent of amount of alcohol consumption, causes continued cell death by apoptosis. Significantly elevated M65 levels in cirrhotic patients after detoxification confirms the continue hepatocyte death by necrosis. Due to the smaller sample size in this study, no levels of significance were reached with regard to M30 differences after detoxification. In contrast to apoptosis markers, TNF-α, IL-6, IL-8 and VEGF were only slightly elevated while no difference was seen with TGF-β in comparison to controls.

In our small sub-cohort with liver histology, only TNF- a correlated significantly with hepatic steatosis, lobular inflammation, ballooning, and fibrosis septa around the central vein while no significant correlations were seen for TGF- b, IL-8 and VEGF. IL-6, IL-8 and VEGF decreased drastically during alcohol withdrawal while TNF- a and TGF- b showed only a moderate decrease. However, when looking at the cirrhotic subcohort, the pro-fibrogenic TGF- b decreased highly significantly reaching almost identical levels in both subgroups. This is in line with other reports where statistically significant reductions in TGF b after the detoxification period show a positive correlation with the reduction of fibrosis (Neuman et al., 2015a,b, 2020).

In the cirrhosis subgroup, cytokine changes were significant in response to alcohol withdrawal. Only VEGF levels remained higher in the cirrhosis group. This could be a first support for the recently introduced sinusoidal pressure hypothesis (SPH) to explain fibrosis progression (Mueller S., 2016). SPH identifies as an elevation of sinusoidal pressure (SP) as cause of fibrosis/cirrhosis. This postulates that elevated SP is the major upstream event that initiates fibrosis progression *via* biomechanical signaling by stretching of perisinusoidal cells. SPH further postulates that arterialization of the stiff cirrhotic liver causes the final self-perpetuating key event exposing the low-pressure-organ to pathologically high pressures. This arterialization, however, is strongly driven by angiogenetic signaling pathways including hypoxia signaling. Since VEGF is one of the major target genes of hypoxia inducible factor 1 alpha, sustained levels of VEGF in cirrhotics during alcohol detoxification could be a first hint that angiogenesis and revascularization is an important process in manifest cirrhosis in support of SPH.

In summary, we here demonstrate important findings on biomarkers of apoptosis, inflammation and angiogenesis in patients with ALD undergoing detoxification. While apoptosis seems to be a hallmark primarily in patients with manifest cirrhosis, it further increases especially in non-cirrhotic patients after alcohol withdrawal. Moreover, both pro-fibrogenic TGF-beta and pro-angiogenic VEGF responded especially effectively in cirrhotics to alcohol withdrawal. These findings seem to be in line with clinical observations and may pave the way to novel targeted therapeutic approaches. Finally, in our opinion, studies on heavy drinkers undergoing alcohol withdrawal represents a valid approach to better understand molecular mechanisms of liver damage and resolution in patients with ALD.

## Data Availability Statement

The original contributions presented in the study are included in the article/Supplementary Material, further inquiries can be directed to the corresponding author/s.

## Ethics Statement

The studies involving human participants were reviewed and approved by Center for Alcohol Research, University of Heidelberg. The patients/participants provided their written informed consent to participate in this study.

## Author Contributions

This work resulted from a collaboration between an academic clinician (SM) and an academic clinical biochemist and toxicologist (MN). MN and SM: substantial contributions to the conception or design of the work acquisition, analysis, and interpretation of data for the work. MN, SM, and JM: writing—original draft preparation, and review and editing. All authors have read and agreed to the published version of the manuscript and are accountable for all aspects of the work in ensuring the accuracy of any part of the work.

## Conflict of Interest

The authors declare that the research was conducted in the absence of any commercial or financial relationships that could be construed as a potential conflict of interest.

## Abbreviations

ALD: alcohol-related liver disease
ACLF: acute-on-chronic liver failure
AH: Alcoholic hepatitis
ALD: Alcohol-related liver disease
ALT: alanine aminotransferase (glutamic-pyruvic transaminase GPT)
ALP: alkaline phosphatase
AMA: anti-mitochondrial antibody
ANA: antinuclear antibody
AST: aspartate aminotransferase (glutamic-oxalic- transaminase GOT)
ASH: alcoholic steatohepatitis
BMI: body mass index
CAP: controlled attenuation parameter
CCK: caspase cleaved cytokeratin (8 and 18)- M30-M 65
DAMPs: danger-associated molecular patterns
EGF: epithelial growth factor
FDA: Food and Drug Administration
GGT: γ-glutamyl-transferase
HBV: hepatitis virus B
HCV: hepatitis virus C
HGF: hepatocyte growth factor
IL: Interleukin: IL-6 IL-8
INR: International Normalized Ratio
Mean ± SD: mean ± standard deviation
PAMPs: pathogen-associated molecular patterns
PRRs: pattern-recognition receptors
TGF-beta: transforming growth factor beta
TNF-α: Tumor necrosis factor.
