# Supplementary material for: Non-invasive Biomarkers of Liver Inflammation and Cell Death in Response to Alcohol Detoxification
Source: Front Physiol. 2021 Jul 7;12:678118. doi: 10.3389/fphys.2021.678118 (PMC8292967; doi:10.3389/fphys.2021.678118)
Supplement: Supplementary file 1 [file Table_1.DOCX]

**Supplemental tables**

**Suppl. Table 1 - Spearman correlation of cytokines with lab and ultrasound n=114**

| **Parameter** | **M30** | **M65** | **TNF-**  **alpha** | **TGF-**  **beta** | **IL-6** | **IL-8** | **VEGF** |
| --- | --- | --- | --- | --- | --- | --- | --- |
| **Sex** | -0.014 | 0.013 | -0.075 | 0.012 | 0.013 | -0.111 | 0.028 |
| **Age** | 0.188* | 0.160 | 0.063 | -0.050 | -0.119 | -0.068 | -0.206* |
| **BMI** | 0.162 | 0.147 | 0.012 | -0.057 | -0.024 | 0.021 | -0.150 |
| **Alcohol consumption** | -0.234* | -0.176 | -0.230* | 0.078 | -0.012 | -0.100 | -0.057 |
| **Duration of heavy alcohol drinking** | -0.039 | -0.027 | -0.016 | 0.036 | -0.027 | -0.081 | 0.008 |
| **M30** | 1.000*** | 0.884*** | 0.147 | -0.072 | -0.010 | 0.076 | -0.234* |
| **M65** | 0.884*** | 1.000*** | 0.130 | -0.053 | 0.006 | 0.104 | -0.306*** |
| **TNF-alpha** | 0.147 | 0.130 | 1.000*** | -0.219* | -0.252** | 0.642*** | 0.254** |
| **TGF-beta** | -0.072 | -0.053 | -0.219* | 1.000*** | 0.193* | -0.245** | 0.180 |
| **IL-6** | -0.010 | 0.006 | -0.252** | 0.193* | 1.000*** | -0.147 | 0.017 |
| **IL-8** | 0.076 | 0.104 | 0.642*** | -0.245** | -0.147 | 1.000*** | 0.345*** |
| **VEGF** | -0.234* | -0.306*** | 0.254** | 0.180 | 0.017 | 0.345** | 1.000*** |
| **AST** | 0.709*** | 0.811*** | 0.085 | -0.089 | -0.067 | -0.031 | -0.307*** |
| **ALT** | 0.451*** | 0.504*** | -0.010 | -0.065 | -0.044 | -0.128 | -0.173 |
| **GGT** | 0.586*** | 0.691*** | 0.071 | -0.090 | 0.043 | 0.121 | -0.265** |
| **AP** | 0.441*** | 0.461*** | 0.235* | -0.142 | -0.001 | 0.144 | 0.012 |
| **Bilirubin total** | 0.459*** | 0.523*** | 0.301** | -0.232* | 0.038 | 0.241* | -0.134 |
| **Quick** | -0.416*** | -0.439*** | -0.307*** | 0.210* | 0.080 | -0.284** | 0.097 |
| **INR** | 0.435*** | 0.434*** | 0.174 | -0.150 | 0.017 | 0.197* | -0.111 |
| **Urea** | -0.287** | -0.288** | 0.018 | -0.030 | -0.047 | -0.001 | -0.003 |
| **Creatinine** | -0.096 | -0.105 | -0.033 | 0.046 | 0.097 | 0.038 | -0.055 |
| **Lipase** | 0.282** | 0.315*** | 0.171 | -0.148 | 0.005 | 0.091 | -0.137 |
| **PTT** | 0.432*** | 0.352*** | 0.052 | -0.069 | 0.160 | 0.000 | -0.113 |
| **Hemoglobin** | -0.200* | -0.146 | -0.299** | 0.095 | 0.051 | -0.203* | 0.009 |
| **Hematocrite** | -0.214* | -0.165 | -0.278** | 0.068 | 0.044 | -0.177 | 0.030 |
| **MCV** | 0.374*** | 0.421*** | 0.245** | -0.044 | -0.156 | 0.065 | -0.174 |
| **Erythrocytes** | -0.356*** | -0.340*** | -0.318*** | 0.065 | 0.118 | -0.153 | 0.115 |
| **Leukocytes** | -0.149 | -0.169 | 0.199* | 0.081 | -0.007 | 0.139 | 0.198* |
| **Sodium** | -0.103 | -0.181 | -0.232* | 0.132 | 0.013 | -0.181 | -0.076 |
| **Potassium** | -0.006 | -0.081 | -0.068 | -0.033 | -0.081 | 0.070 | -0.002 |
| **Platelets** | -0.292** | -0.337*** | -0.007 | 0.202* | -0.028 | 0.052 | 0.382*** |
| **Ferritin** | 0.416*** | 0.451*** | 0.238* | -0.277** | -0.111 | 0.147 | -0.245** |
| **CRP** | 0.326*** | 0.326*** | 0.391*** | -0.017 | 0.022 | 0.231* | -0.005 |
| **Liver stiffness** | 0.567*** | 0.604*** | 0.168 | -0.111 | -0.012 | 0.145 | -0.133 |
| **CAP** | 0.169 | 0.179 | -0.095 | 0.082 | 0.273 | 0.050 | -0.073 |
| **Liver size** | 0.257* | 0.348*** | -0.048 | 0.063 | 0.022 | 0.018 | -0.154 |
| **Hepatic steatosis (US)** | 0.247* | 0.340** | 0.012 | -0.079 | 0.032 | 0.030 | -0.271** |
| **Spleen size** | 0.362*** | 0.315** | 0.143 | -0.312** | -0.040 | 0.089 | -0.216* |
| **Ascites** | 0.306** | 0.339*** | 0.288** | -0.061 | -0.017 | 0.258** | 0.050 |
| **Signs of cirrhosis (US)** | 0.430*** | 0.452*** | 0.235* | -0.144 | 0.046 | 0.184 | -0.126 |

*: P<0.05, **: P<0.01, **: P<0.001

**Suppl. Table 2 - Spearman correlations of histology with apoptosis markers and cytokines**

| **Spearman** | **M30** | **M65** | **TNF-alpha** | **TGF-beta** | **IL-6** | **IL-8** | **VEGF** |
| --- | --- | --- | --- | --- | --- | --- | --- |
| **Kleiner steatosis 0-3** | 0.092 | 0.269 | 0.415* | -0.049 | -0.544** | 0.156 | -0.204 |
| **location 0-3** | -0.114 | 0.161 | 0.054 | -0.110 | -0.385 | -0.052 | -0.303 |
| **microvesicular 0-1** | 0.228 | 0.359 | 0.290 | -0.032 | -0.129 | -0.064 | 0.354 |
| **lobular inflammation 0-3** | 0.506* | 0.489* | 0.441* | -0.104 | -0.292 | 0.334 | 0.159 |
| **microgranulomas 0-1** | 0.167 | 0.318 | 0.015 | 0.188 | -0.142 | 0.164 | -0.246 |
| **large lipogranulomas 0-1** | 0.204 | 0.318 | 0.329 | 0.128 | -0.164 | 0.157 | -0.007 |
| **portal inflammation 0-1** | 0.138 | 0.197 | 0.156 | 0.186 | -0.166 | 0.117 | 0.068 |
| **ballooning 0-2** | 0.419* | 0.449* | 0.557** | -0.113 | -0.432* | 0.366 | 0.136 |
| **acidophil bodies 0-1** | 0.220 | 0.346 | 0.046 | 0.066 | -0.440* | -0.007 | -0.085 |
| **pigmented macrophages 0-1** | 0.042 | 0.000 | 0.304 | -0.325 | -0.172 | 0.048 | -0.234 |
| **megamitochondria 0-1** | 0.089 | 0.161 | -0.128 | 0.064 | -0.294 | -0.342 | 0.143 |
| **mallory hyaline 0-1** | 0.575** | 0.651*** | 0.24 | 0.128 | -0.052 | 0.314 | 0.269 |
| **glycogenated nuclei 0-1** | -0.210 | -0.303 | -0.021 | 0.122 | 0.328 | -0.028 | -0.214 |
| **classification steatohepatitis 0-2** | 0.340 | 0.510* | 0.466* | 0.101 | -0.415* | 0.302 | -0.044 |
| **0-4 A-C** | 0.152 | 0.146 | 0.327 | -0.159 | -0.171 | 0.013 | -0.163 |
| **Fibrosis stage 0-4** | 0.301 | 0.398 | 0.292 | 0.204 | -0.271 | 0.239 | -0.062 |
| **CLV** | 0.269 | 0.385 | 0.487* | -0.183 | -0.580** | 0.383 | 0.137 |
| **PS** | 0.179 | 0.246 | 0.34 | 0.044 | -0.317 | 0.109 | -0.044 |
| **PT** | 0.144 | 0.115 | 0.105 | 0.311 | 0.003 | 0.051 | -0.033 |
| **Septa** | 0.294 | 0.336 | 0.264 | 0.201 | -0.165 | 0.169 | 0.029 |
| **WS** | 0.252 | 0.257 | 0.279 | 0.229 | -0.185 | 0.162 | 0.001 |
| **Chevallier-Score (SSS)** | 0.258 | 0.283 | 0.295 | 0.188 | -0.215 | 0.186 | 0.008 |

*: P<0.05, **: P<0.01, **: P<0.001

**Suppl. Table 3A - Spearman correlations of detox changes of apoptotic markers and cytokines with initial levels of clinical parameters (n=45)**

| **Before Detox** | **ΔM30** | **ΔM65** | **ΔTNF- alpha** | **ΔTGF- beta** | **ΔIL-6** | **ΔIL-8** | **ΔVEGF** |
| --- | --- | --- | --- | --- | --- | --- | --- |
| **M30** | -0.345* | -0.398* | -0.185 | 0.010 | -0.071 | -0.285 | 0.013 |
| **M65** | -0.508** | -0.433* | -0.198 | -0.069 | -0.093 | -0.368* | 0.104 |
| **TNF-alpha** | -0.424* | -0.345* | -0.623*** | 0.269 | 0.304* | -0.605*** | -0.316* |
| **TGF-beta** | -0.048 | 0.132 | 0.005 | -0.794*** | -0.205 | 0.129 | -0.213 |
| **IL-6** | 0.198 | -0.095 | 0.040 | -0.391** | -0.598*** | 0.327* | 0.059 |
| **IL-8** | -0.486** | -0.222 | -0.299* | 0.179 | 0.305* | -0.849*** | -0.208 |
| **VEGF** | -0.097 | 0.053 | -0.202 | -0.037 | 0.643*** | -0.231 | -0.838*** |
| **AST** | -0.298 | -0.330 | -0.111 | -0.030 | -0.180 | -0.137 | 0.026 |
| **ALT** | 0.068 | 0.055 | 0.056 | 0.037 | -0.026 | 0.132 | -0.104 |
| **GGT** | -0.299 | -0.356* | -0.060 | -0.056 | -0.170 | -0.133 | 0.191 |
| **AP** | -0.201 | -0.330 | -0.076 | 0.036 | 0.081 | -0.221 | 0.120 |
| **Bilirubin total** | -0.249 | -0.255 | -0.277 | 0.074 | 0.052 | -0.376* | 0.067 |
| **Quick** | 0.087 | -0.035 | 0.009 | 0.058 | -0.015 | 0.262 | -0.290 |
| **INR** | -0.013 | 0.045 | 0.024 | -0.084 | 0.001 | -0.186 | 0.287 |
| **Urea** | 0.007 | 0.172 | -0.114 | 0.023 | -0.074 | -0.050 | 0.074 |
| **Creatinine** | -0.001 | -0.103 | -0.004 | -0.008 | -0.118 | -0.102 | 0.143 |
| **Lipase** | -0.164 | -0.143 | -0.087 | 0.128 | -0.203 | -0.081 | 0.003 |
| **PTT** | -0.103 | -0.087 | 0.039 | -0.131 | -0.086 | -0.091 | 0.163 |
| **Hemoglobin** | 0.350* | 0.301 | 0.260 | -0.030 | 0.084 | 0.171 | -0.137 |
| **Hematocrite** | 0.274 | 0.230 | 0.158 | -0.014 | 0.079 | 0.122 | -0.187 |
| **MCV** | -0.034 | -0.194 | -0.127 | -0.005 | -0.077 | -0.082 | 0.289 |
| **Erythrocytes** | 0.220 | 0.213 | 0.190 | -0.003 | 0.036 | 0.136 | -0.229 |
| **Leukocytes** | -0.257 | -0.188 | -0.308* | 0.025 | 0.329* | -0.333* | -0.189 |
| **Sodium** | 0.385* | 0.330 | 0.110 | -0.063 | -0.328* | 0.307* | 0.164 |
| **Potassium** | 0.052 | -0.078 | 0.294 | -0.015 | -0.030 | -0.018 | 0.057 |
| **Platelets** | -0.352* | -0.305 | -0.128 | -0.072 | 0.158 | -0.209 | -0.341* |
| **Ferritin** | -0.244 | -0.287 | -0.057 | 0.170 | -0.069 | -0.094 | 0.216 |
| **CRP** | -0.137 | -0.209 | -0.166 | -0.156 | -0.034 | -0.215 | 0.130 |
| **Liver stiffness** | -0.219 | -0.210 | -0.136 | -0.061 | 0.017 | -0.247 | 0.173 |
| **CAP** | 0.201 | 0.068 | -0.018 | -0.015 | -0.144 | 0.064 | 0.181 |

*: P<0.05, **: P<0.01, **: P<0.001

**Suppl. Table 3B - Spearman correlations of detox changes of apoptotic markers and cytokines with changes of clinical parameters (n=45)**

| **Spearman** |  | |  | |  | |  | |  | |  | |  |
| --- | --- | --- | --- | --- | --- | --- | --- | --- | --- | --- | --- | --- | --- |
|  | **ΔM30** | **ΔM65** | | **ΔTNF- alpha** | | **ΔTGF- beta** | | **ΔIL-6** | | **ΔIL-8** | | **ΔVEGF** | |
| **ΔM30** |  | 0.741*** | | 0.502** | | 0.046 | | -0.136 | | 0.701*** | | 0.341* | |
| **ΔM65** | 0.741*** |  | | 0.423* | | -0.175 | | 0.076 | | 0.432* | | 0.207 | |
| **ΔTNF-alpha** | 0.502** | 0.423* | |  | | -0.121 | | -0.113 | | 0.454** | | 0.351* | |
| **ΔTGF-beta** | 0.046 | -0.175 | | -0.121 | |  | | 0.310 | | -0.162 | | 0.076 | |
| **ΔIL-6** | -0.136 | 0.076 | | -0.113 | | 0.310 | |  | | -0.294 | | -0.428** | |
| **ΔIL-8** | 0.701*** | 0.432* | | 0.454** | | -0.162 | | -0.294 | |  | | 0.316* | |
| **ΔVEGF** | 0.341* | 0.207 | | 0.351* | | 0.076 | | -0.428** | | 0.316* | |  | |
| **ΔAST** | 0.334 | 0.581*** | | 0.120 | | -0.095 | | 0.033 | | 0.186 | | 0.046 | |
| **ΔALT** | 0.324 | 0.529** | | 0.293 | | -0.127 | | -0.285 | | 0.093 | | 0.389** | |
| **ΔGGT** | 0.367* | 0.35* | | 0.106 | | 0.000 | | 0.121 | | 0.334* | | -0.159 | |
| **ΔAP** | 0.231 | 0.264 | | 0.010 | | 0.054 | | 0.199 | | 0.094 | | -0.116 | |
| **ΔBilirubin total** | 0.208 | 0.110 | | 0.232 | | -0.013 | | -0.427** | | 0.129 | | 0.232 | |
| **ΔQuick** | 0.002 | 0.187 | | 0.259 | | -0.013 | | 0.195 | | -0.098 | | 0.155 | |
| **ΔINR** | 0.053 | -0.126 | | -0.130 | | 0.021 | | -0.134 | | 0.091 | | -0.130 | |
| **ΔUrea** | 0.459** | 0.226 | | 0.413** | | -0.212 | | -0.180 | | 0.501*** | | 0.056 | |
| **ΔCreatinine** | 0.246 | 0.136 | | 0.226 | | 0.087 | | -0.089 | | 0.411** | | 0.124 | |
| **ΔLipase** | 0.225 | 0.329 | | 0.247 | | 0.116 | | 0.141 | | 0.031 | | 0.100 | |
| **ΔPTT** | 0.070 | -0.120 | | -0.282 | | 0.200 | | 0.255 | | 0.051 | | -0.149 | |
| **ΔHemoglobin** | 0.408* | 0.171 | | -0.031 | | 0.087 | | -0.189 | | 0.221 | | 0.337* | |
| **ΔHematocrite** | 0.414* | 0.223 | | 0.176 | | 0.113 | | -0.161 | | 0.217 | | 0.333* | |
| **ΔMCV** | -0.245 | -0.010 | | 0.024 | | 0.062 | | -0.029 | | -0.018 | | 0.106 | |
| **ΔErythrocytes** | 0.335 | 0.105 | | 0.104 | | 0.135 | | -0.136 | | 0.207 | | 0.34* | |
| **ΔLeukocytes** | 0.185 | 0.081 | | 0.536*** | | 0.106 | | -0.224 | | 0.256 | | 0.270 | |
| **ΔSodium** | -0.384 | -0.177 | | 0.096 | | -0.078 | | 0.079 | | -0.012 | | -0.089 | |
| **ΔPotassium** | 0.099 | 0.193 | | 0.189 | | 0.081 | | 0.219 | | -0.078 | | 0.086 | |
| **ΔPlatelets** | -0.039 | -0.256 | | 0.057 | | 0.280 | | -0.133 | | 0.057 | | 0.191 | |
| **ΔFerritin** | 0.511** | 0.536** | | 0.233 | | -0.130 | | 0.013 | | 0.345* | | -0.072 | |
| **ΔCRP** | 0.125 | -0.076 | | 0.143 | | 0.283 | | 0.020 | | 0.185 | | 0.053 | |
| **ΔLiver stiffness** | 0.075 | 0.178 | | 0.160 | | -0.177 | | 0.113 | | -0.180 | | -0.046 | |
| **ΔCAP** | 0.012 | -0.012 | | 0.038 | | 0.179 | | 0.177 | | 0.043 | | 0.256 | |

*: P<0.05, **: P<0.01, **: P<0.001

**Suppl. Table 4 - Comparison of cytokines between cirrhotics (n=27) vs non cirrhotics (n=86)**

| **Parameter** | **F0-3**  **N=86** | **F4**  **N=27** | **P** |
| --- | --- | --- | --- |
| **Sex (male)** | 69% | 70% | 0.8641 |
| **Age (years)** | 48.6 ± 12.9 | 54.4 ± 9.1 | 0.0325 |
| **BMI (kg/m^2^)** | 25.8 ± 5.3 | 26.8 ± 4.8 | 0.4223 |
| **Alcohol consumption (g/day)** | 207.9 ± 134.1 | 189.1 ± 192.7 | 0.5753 |
| **Duration of heavy alcohol drinking (years)** | 14.3 ± 10.3 | 20.1 ± 12.6 | 0.0411 |
| **M30 (U/L)** | 434 ± 473 | 949.1 ± 820.9 | <0.0001 |
| **M65 (U/L)** | 851 ± 864 | 1851.6 ± 1156.7 | <0.0001 |
| **TNF-alpha (pg/mL)** | 42.7 ± 40.4 | 51.8 ± 36.0 | 0.2944 |
| **TGF-beta (ng/mL)** | 27.8 ± 26.2 | 21.2 ± 9.8 | 0.2085 |
| **IL-6 (pg/mL)** | 45.3 ± 31.9 | 54.6 ± 35.2 | 0.2037 |
| **IL-8 (pg/mL)** | 54.6 ± 50.7 | 96.5 ± 111.4 | 0.0076 |
| **VEGF (pg/mL)** | 81.0 ± 50.9 | 70.7 ± 47.3 | 0.3503 |
| **AST (U/L)** | 103 ± 100 | 136 ± 52 | 0.1091 |
| **ALT (U/L)** | 76 ± 61 | 69 ± 83 | 0.6621 |
| **GGT (U/L)** | 403 ± 613 | 793 ± 834 | 0.0102 |
| **AP (U/L)** | 95 ± 43 | 173 ± 94 | <0.0001 |
| **Bilirubin total (mg/dL)** | 1.03 ± 2.07 | 3.60 ± 4.52 | <0.0001 |
| **Quick (%)** | 109.3 ± 16.9 | 76.3 ± 21.7 | <0.0001 |
| **INR** | 0.93 ± 0.24 | 1.21 ± 0.23 | <0.0001 |
| **Urea (mg/dL)** | 23.5 ± 19.7 | 18.8 ± 12.3 | 0.2439 |
| **Creatinine (mg/dL)** | 0.71 ± 0.24 | 0.67 ± 0.34 | 0.5241 |
| **Lipase (U/L)** | 60.3 ± 114.4 | 60.0 ± 36.6 | 0.9891 |
| **PTT (sec)** | 32.0 ± 7.1 | 37.8 ± 5.9 | 0.0007 |
| **Hemoglobin (g/dL)** | 14.1 ± 1.7 | 12.7 ± 2.2 | 0.0008 |
| **Hematocrite (%)** | 39.7 ± 4.7 | 35.9 ± 6.2 | 0.0009 |
| **MCV (fL)** | 93.8 ± 15.4 | 99.9 ± 8.3 | 0.0548 |
| **Erythrocytes (/pL)** | 4.3 ± 0.7 | 3.6 ± 0.8 | <0.0001 |
| **Leukocytes (/nL)** | 7.7 ± 2.6 | 7.9 ± 3.4 | 0.8173 |
| **Sodium(mmol/L)** | 137.4 ± 4.9 | 136.2 ± 4.9 | 0.2445 |
| **Potassium(mmol/L)** | 3.8 ± 0.4 | 3.7 ± 0.5 | 0.2208 |
| **Platelets (/nL)** | 206.4 ± 76.9 | 160.9 ± 83.0 | 0.0098 |
| **Ferritin (ng/mL)** | 678 ± 670.7 | 925.4 ± 815.6 | 0.1156 |
| **CRP (mg/L)** | 7.9 ± 16.9 | 12.5 ± 14.4 | 0.2052 |
| **Liver stiffness (kPa)** | 8.3 ± 8.2 | 60.1 ± 14.6 | <0.0001 |
| **CAP (dB/m)** | 306 ± 50 | 297 ± 54 | 0.6260 |
| **Liver size (cm)** | 16.0 ± 3.7 | 17.6 ± 3.3 | 0.0501 |
| **Hepatic steatosis (US, 0-3)** | 1.80 ± 0.85 | 1.85 ± 0.90 | 0.8659 |
| **Spleen size (cm)** | 10.0 ± 2.7 | 11.9 ± 2.2 | 0.0039 |
| **Ascites (US, %)** | 1% | 35% | <0.0001 |
| **Signs of cirrhosis (US, %)** | 2% | 80% | <0.0001 |
